# Cigarette smoke downregulates Nur77 to exacerbate inflammation in chronic obstructive pulmonary disease (COPD)

**DOI:** 10.1371/journal.pone.0229256

**Published:** 2020-02-21

**Authors:** Aravind T. Reddy, Sowmya P. Lakshmi, Asoka Banno, Shantanu Krishna Jadhav, Ishaque Pulikkal Kadamberi, Seong C. Kim, Raju C. Reddy

**Affiliations:** 1 Department of Medicine, Division of Pulmonary, Allergy and Critical Care Medicine, University of Pittsburgh School of Medicine, Pittsburgh, Pennsylvania, United States of America; 2 Veterans Affairs Pittsburgh Healthcare System, Pittsburgh, Pennsylvania, United States of America; Istituti Clinici Scientifici Maugeri, ITALY

## Abstract

Cigarette smoke (CS) contains multiple gaseous and particulate materials that can cause lung inflammation, and smoking is the major cause of chronic obstructive pulmonary disease (COPD). We sought to determine the mechanisms of how CS triggers lung inflammation. Nur77, a nuclear hormone receptor belonging to the immediate-early response gene family, controls inflammatory responses, mainly by suppressing the NF-κB signaling pathway. Because it is unknown if Nur77’s anti-inflammatory role modulates COPD, we assessed if and how Nur77 expression and activity are altered in CS-induced airway inflammation. In lung tissues and bronchial epithelial cells from COPD patients, we found Nur77 was downregulated. In a murine model of CS-induced airway inflammation, CS promoted lung inflammation and also reduced Nur77 activity in wild type (WT) mice, whereas lungs of Nur77-deficient mice showed exaggerated CS-induced inflammatory responses. Our findings in *in vitro* studies of human airway epithelial cells complemented those *in vivo* data in mice, together showing that CS induced threonine-phosphorylation of Nur77, which is known to interfere with its anti-inflammatory functions. In summary, our findings point to Nur77 as an important regulator of CS-induced inflammatory responses and support the potential benefits of Nur77 activation for COPD treatment.

## Introduction

Chronic obstructive pulmonary disease (COPD) is an inflammatory lung disease characterized by persistent airflow limitation and impaired gas exchange [1]. It involves millions of people and is a major socioeconomic burden [2]. Because therapeutic strategies currently available to patients fail to prevent its progression and exacerbations effectively [3], COPD-associated morbidity and mortality are anticipated to increase in the coming years [2]. Cigarette smoking is the primary cause of COPD, and many gaseous and particulate materials contained within first- and second-hand cigarette smoke (CS) can cause lung inflammation [4–8]. Nevertheless, current understanding of how CS drives lung inflammation, such as that associated with COPD, remains incomplete [9].

To discover mechanistic insights into the molecular pathophysiology of CS-induced lung inflammation and COPD, in this study, we tested the potential mediating roles and actions of Nur77 [10, 11] (also known as NR4A1), a specific member of the immediate-early response gene family. Together with Nur-related factor 1 (Nurr1 [12]; also known as NR4A2) and neuron-derived orphan receptor 1 (NOR-1 [13]; also known as NR4A3), it forms the NR4A subfamily of nuclear hormone receptors [14, 15]. One unique feature of these transcription factors is the atypical ligand-binding domain; crystallographic studies have demonstrated bulky hydrophobic ligand-binding pockets in the Nurr1 ligand-binding domain [16] and the rat Nur77 ligand-binding domain [17]. These earlier findings combined with the failure to locate endogenous ligands had classified NR4A members as orphan nuclear receptors [16, 17]. More recent studies, however, have revealed that small synthetic molecules [18, 19] and some unsaturated fatty acids [20, 21] can bind to and modulate Nur77.

Nur77 plays a role in a number of biological and pathophysiological processes [15, 22]. Accumulating evidence indicates that it regulates multiple inflammation-related conditions [23], primarily mediated via its effects on the NF-κB signaling pathway [19, 24–28]. In the respiratory system, Nur77 dampened OVA-induced airway inflammation in the murine model of allergic airway disease [26]. Nur77 also controlled inflammatory responses and prevented the resulting tissue damage in a rat model of acute respiratory distress syndrome [27]. Furthermore, microarray studies coupled with gene set enrichment analyses and Ingenuity pathway analyses revealed an association of Nur77 with COPD and allergic airway inflammatory disease, respectively [29]. Nonetheless, evidence defining specific anti-inflammatory functions of Nur77 in COPD is scarce.

Therefore, using multiple approaches, we tested the hypothesis that Nur77 contributes to CS-induced airway inflammation associated with COPD. We found that lung tissues from COPD patients displayed reduced Nur77 expression. Similarly, both Nur77 expression and its transcriptional activity were reduced in human bronchial epithelial (HBE) cells from COPD patients (COPD HBE cells), suggesting a link between Nur77 downregulation and COPD pathogenesis. Furthermore, we found that CS downregulated Nur77 expression and activity and also exacerbated inflammatory responses, both in mice *in vivo* and in human airway epithelial cells *in vitro*. Our finding that CS-induced Nur77 suppression aggravates airway epithelial inflammation suggests the possibility that treatment with Nur77 agonists may be a useful therapeutic tool to counteract pro-inflammatory effects of cigarette smoking in COPD.

## Materials and methods

### Cells, tissue samples, and treatments

HBE cells (normal HBE [NHBE] and diseased HBE [DHBE; also described as COPD HBE]) obtained from Lonza (Walkersville, MD) were grown and maintained in bronchial epithelial cell medium (Lonza) supplemented with growth factors and hormones, according to the manufacturer’s instructions. H292 cells were obtained from ATCC (Rockville, MD) and maintained in RPMI medium supplemented with FBS, penicillin, and streptomycin (ThermoFisher Scientific; Waltham, MA). Cells were cultured at 37°C in a humidified atmosphere of 5% CO_2_. Monolayer cultures at 90% confluence were deprived of growth factors before treatment.

Human lung tissue sampling was done as reported previously [30]. COPD lung tissues were obtained from explanted lungs of subjects with advanced COPD, and control lungs were donated lungs not suitable for transplantation, obtained from the Center for Organ Recovery and Education. Lung tissues were stored at −80°C until future usage.

Cytosporone B (Csn-B [C2997; Sigma-Aldrich, St. Louis, MO]), SB203580 (5633; Cell Signaling Technology, Beverly, MA) and PD98059 (9900; Cell Signaling Technology) were dissolved in DMSO to prepare stock solutions (100 mM). Working concentrations were prepared by further dilution with DMSO. Cells deprived of growth factors were treated with the indicated concentrations as previously described [30–32].

### Animals

C57BL/6 (000664; wild type [WT]) and *Nr4a1* (Nur77) knockout (KO) mice (006187) [33] were obtained from the Jackson Laboratories (Bar Harbor, ME). Mice were housed in microisolator cages under specific pathogen-free conditions and fed autoclaved food (Teklad global 18% protein rodent diet; Envigo [Hackensack, NJ]). Male mice aged 6–8 weeks (20–25 g) were used in all experiments. Mice were euthanized by exposure to CO_2_ in a flow-controlled CO_2_ chamber followed by cervical dislocation or post-mortem sample collection. All studies were performed according to a protocol reviewed and approved by the VA Pittsburgh Healthcare System Institutional Animal Care and Use Committee (protocol #03028).

### CSE preparation and exposure

Cigarette smoke extract (CSE) medium was prepared as described previously [30]. Briefly, air or smoke from research-grade cigarettes (3R4F; Kentucky Tobacco Research and Development Center, University of Kentucky, Lexington, KY) was slowly bubbled into 10 ml of cell culture medium according to the Federal Trade Commission protocol, with one puff for 2 seconds. The medium was then sterilized using a 0.22-μm filter (EMD Millipore, Billerica, MA). A portion of the CSE medium was used to measure its optical density at 320 nm. The extract, defined as 100% CSE, was diluted to the indicated concentrations and used within 10 minutes of preparation.

### CS exposure

Mice were exposed to CS or to filtered air for two months as described previously [34]. Briefly, CS was generated by burning five 3RF4 research cigarettes according to the Federal Trade Commission protocol, each puff being of 2 second duration at a flow rate of 1.05 l/min and 35 ml volume, in an automated TE-10 smoking machine (Teague Enterprises, Davis, CA). The machine was adjusted to produce 89% sidestream and 11% mainstream smoke. The chamber atmosphere was monitored to maintain TPM at 250 mg/m^3^. Twenty-four hours following the last exposure, mice were euthanized, and the lungs were collected for further analysis.

### BAL fluid collection and cell count

Bronchoalveolar lavage (BAL) fluid was collected by flushing 1 ml of PBS containing 0.1 mM EDTA into the lung via a tracheal cannula three times. After the pooled BAL fluid was centrifuged, cells were pelleted and then resuspended in 1 ml of PBS. The number of total cells was counted using a hemocytometer. Cytospin preparations were stained with Diff-Quik (Siemens, Newark, DE).

### Western blotting

Protein concentrations were determined using the BCA Protein Assay kit (ThermoFisher Scientific). Western blotting was then performed as described previously [32]. Primary antibodies against pThr (5267) and β-Actin (1616) were purchased from Santa Cruz Biotechnology (Santa Cruz, CA). Anti-Nur77 antibody was from Abcam (109180; Cambridge, MA). The secondary antibodies, donkey anti-goat IRDye 680RD (926–68074) and goat anti-rabbit IRDye 800CW (925–32211), were obtained from LI-COR Biosciences (Lincoln, NE). The infrared signal was detected with an Odyssey Infrared Imager (LI-COR Biosciences). The densitometric analysis was performed using Image Studio software (LI-COR Biosciences).

### Immunostaining for Nur77

Immunostaining was performed on lung tissue sections to detect Nur77 expression as described previously [35]. Lung sections were obtained from GeneTex (#24349 and 21848, at 5 μm thickness mounted on positively charged glass slides; Irvine, CA). Briefly, the lung sections were deparaffinized in xylene and then rehydrated in a series of graded alcohols. Sections were then permeabilized with target retrieval solution. Endogenous peroxidase was blocked with 3% hydrogen peroxide for 10 min. Staining was performed with anti-Nur77 antibody (109180; Abcam). VECTASTAIN Elite ABC HRP Kit (PK-6101) and DAB Peroxidase (HRP) Substrate Kit (SK-4100) from Vector Laboratories (Burlingame, CA) were used for secondary antibody binding and for color development, respectively. Control sections were incubated with an isotype-matched rabbit IgG (2729; Cell Signaling). Slides were counterstained with hematoxylin and imaged.

### Electrophoretic mobility shift assay

Nuclear extracts from NHBE cells were incubated with 50 nM of the indicated double-stranded oligonucleotides (S1 Table) 5´end-labeled with infrared dye IRDye 700 in binding buffer (100 mM Tris, 500 mM KCL, 10 mM DTT [pH 7.5]), poly (deoxyinosinic-deoxycytidylic) (1 μg/μl in 10 mM Tris, 1 mM EDTA), 25 mM DTT, and 2.5% Tween 20. Samples were then separated on 5% non-denaturing polyacrylamide gels in 1× Tris-Borate EDTA buffer (130 mM Tris [pH 8.3], 45 mM boric acid, 2.5 mM EDTA). In the supershift assay, the reaction mixture was incubated with anti-Nur77 antibody (365113; Santa Cruz Biotechnology). The infrared signal was detected using an Odyssey Infrared Imager.

### Measurement of cytokine, chemokine and transcription factor activity

ELISA-based cytokine and chemokine measurements (DTA00D [human tumor necrosis factor-α, TNFα], D8000C [human interleukin 8, IL-8], MTA00B [mouse TNFα], M6000B [mouse interleukin 6, IL-6], MJE00B [mouse monocyte chemoattractant protein-1, MCP-1], and MKC00B [mouse KC]; R&D Systems, Minneapolis, MN) and ELISA-based transcription factor-DNA binding assay (40096 [NF-κB p65]; Active Motif, Carlsbad, CA) were done according to the manufacturer’s instructions and as described previously [30, 31].

### Nur77-NurRE binding assay

Biotinylated Nur-response element (NurRE) or nonspecific oligonucleotides (S1 Table) were coated onto streptavidin-coated 96 well plates (Pierce) and incubated at room temperature for 1 h. After incubation, the wells were emptied and washed with 200 μl PBST (PBS supplemented with 0.05% Tween-20) three times to remove the unbound oligos. Protein samples were added to the wells and incubated at room temperature for 1 h. Unbound proteins were then removed by washing with 200 μl PBST three times. Nur77 binding was detected using anti-Nur77 antibody, and the infrared signal was read with an Odyssey Infrared Imager. Nur77-NurRE binding is represented as the normalized signal and compared with experimental controls.

### RNA isolation, nascent RNA capturing, and real-time PCR

RNA isolation, cDNA synthesis, and real-time PCR were performed as described previously [7]. Briefly, total RNA was isolated using the RNeasy Plus Mini Kit (Qiagen, Valencia, CA), and cDNA was generated from total RNA using iScript Advanced cDNA Synthesis Kit (#1725038; Bio-Rad, Hercules, CA). Real-time PCR was then performed with specific primers for Nur77 and β-Actin (S1 Table) and Fast SYBR Green Master Mix (Applied Biosystems, Foster City, CA). Relative expression normalized to β-Actin is presented.

### Statistical analysis

Data are presented as the mean ± SD. We determined the differences between experimental groups using an unpaired *t*-test or one-way or two-way analysis of variance followed by a Bonferroni multiple-comparison correction. For statistical analyses, we used GraphPad Prism 8.1.2 (GraphPad Software, La Jolla, CA); differences with *P* values < 0.05 were considered significant.

## Results

### Reduced expression and altered distribution of Nur77 in lung tissues of COPD patients

**Fig 1:**
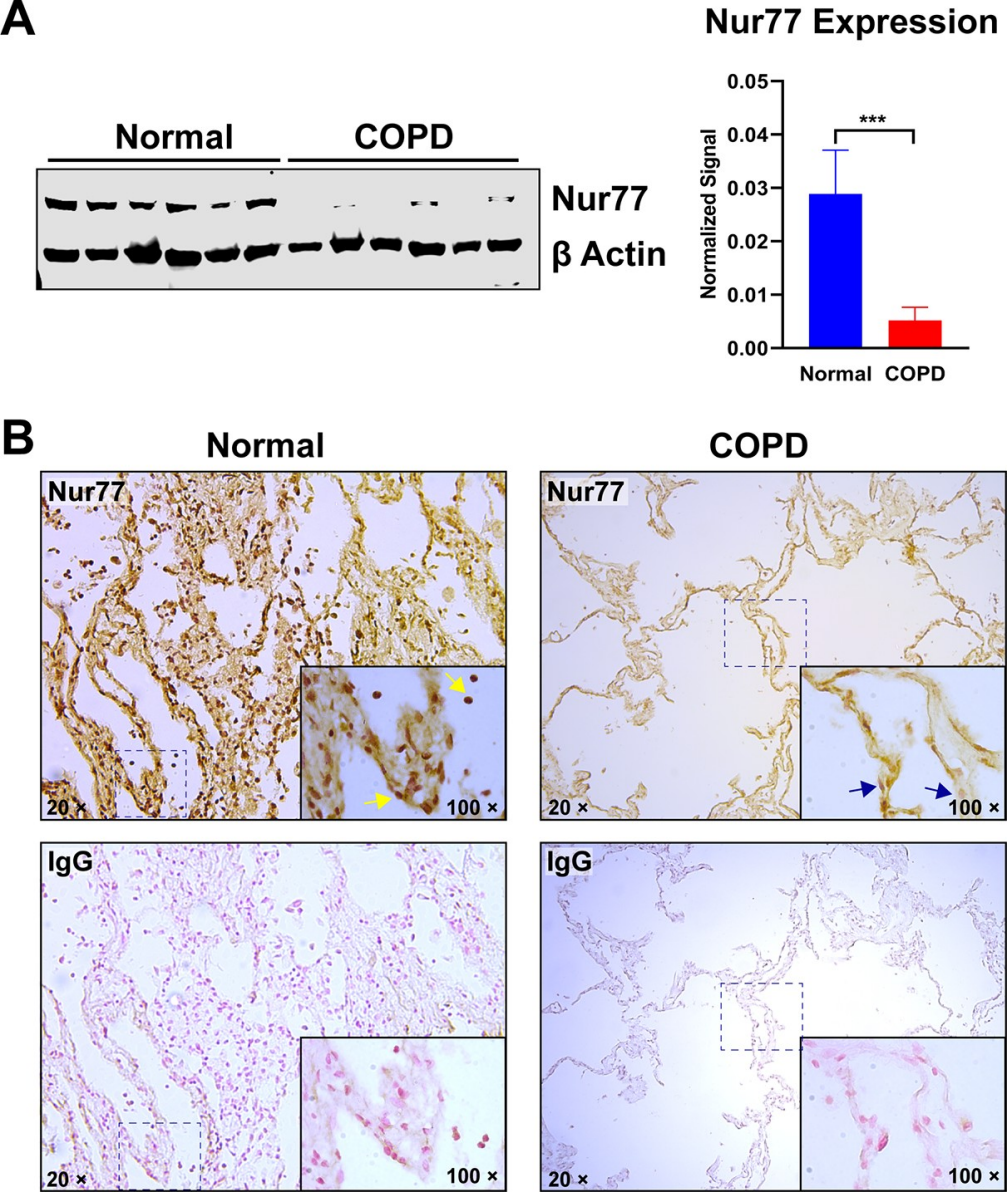
Nur77 expression is reduced in COPD. (**A**) Nur77 expression in normal human lung tissues and those of COPD patients, determined by Western blotting and densitometric analysis. β-Actin served as a loading control. (**B**) Nur77 expression in COPD and normal human lung tissues, determined by immunostaining. Paraffin sections were immunostained with anti-Nur77 antibody (*upper panels*) or IgG negative control (*lower panels*). Cells/nuclei positive for Nur77 signal are indicated by *yellow arrows*. Cells/nuclei in COPD lung tissue showing reduced Nur77 signal are marked with *blue arrows*. Representative images at the indicated magnification (100× or 20×) are shown. COPD patient tissue (*right panels*) also show characteristic loss of alveolar structure. Data are expressed as the mean ± SD with *n* = 6; ***P < 0.001.

Anti-inflammatory actions of Nur77 have been described in several pathophysiological systems, but understanding of whether it contributes to development of COPD remains limited. Therefore, we tested whether Nur77 expression is altered in lung tissues of COPD patients, by comparing its levels with those in lungs of normal controls. Nur77 protein levels were significantly lower in COPD lung tissues than in normal human lung tissue (Fig 1A) and were undetectable in some patients (Fig 1A). Similarly, by immunostaining analysis, we detected Nur77 in nearly all cells in normal human lungs but found it was diminished in COPD lung tissues (Fig 1B). Also, Nur77 accumulated within cell nuclei in normal lung tissues (Fig 1B, *yellow arrows*), but such nuclear accumulation was reduced in COPD lung tissues (Fig 1B, *blue arrows*). These results suggest that Nur77 downregulation may be associated with human COPD pathogenesis.

### Nur77 expression and activity are reduced in HBE cells of patients with COPD

**Fig 2:**
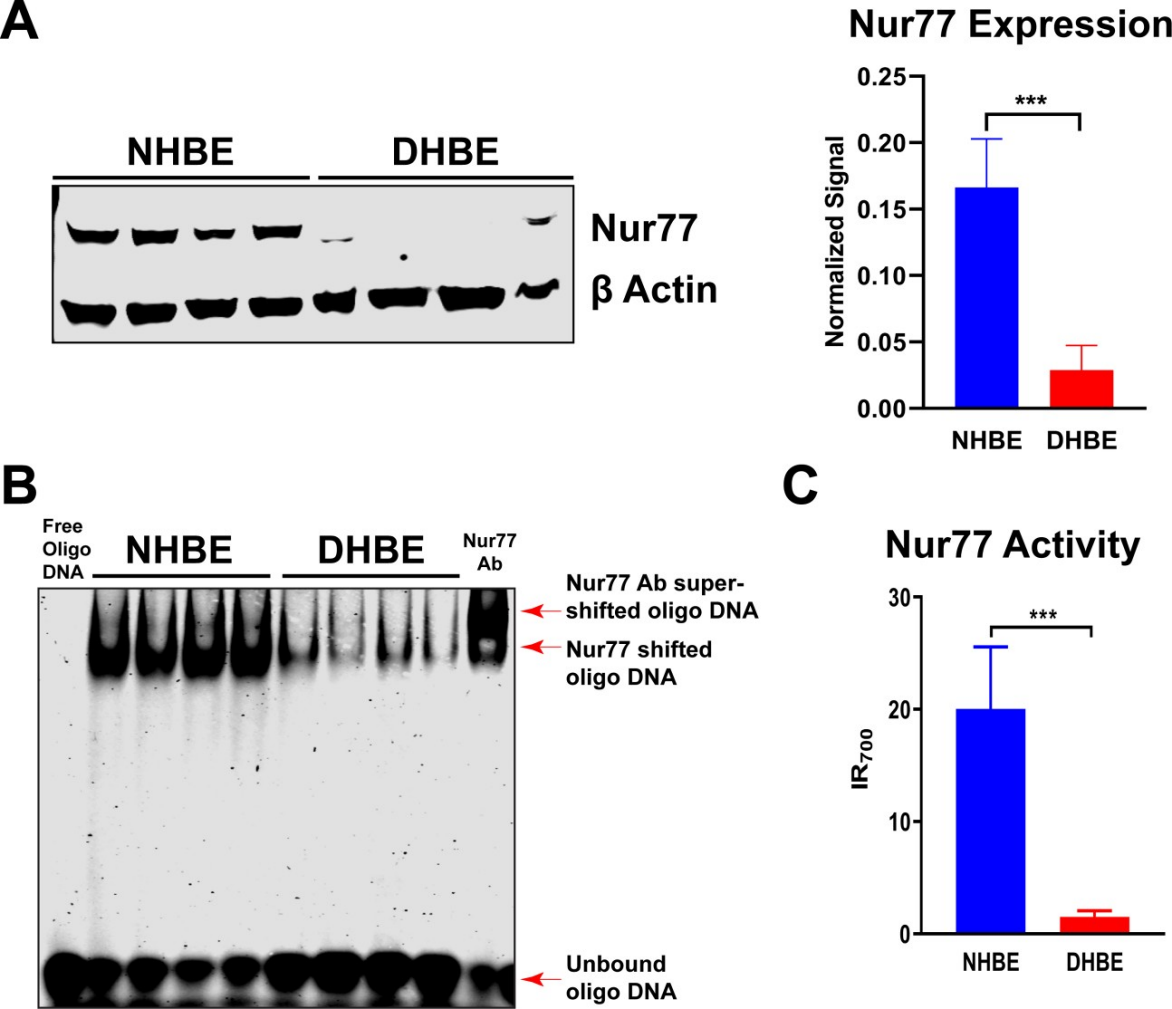
Nur77 expression and activity are decreased in HBE cells of COPD patients. (**A**) Nur77 expression in DHBE (also described as COPD HBE in the text) and NHBE cells, determined by Western blotting and densitometric analysis. β-Actin served as a loading control. (**B**) Nur77 NurRE binding in NHBE and DHBE cell nuclear extracts, determined by electrophoretic mobility shift assay. The supershift of the Nur77-NurRE complex in the presence of anti-Nur77 antibody confirmed the specificity of this interaction. (**C**) DNA-binding activity of Nur77 in NHBE and DHBE cells, determined by Nur77-specific reporter assay. Shown is infrared assay endpoint signal level (IR_700_). Data are expressed as the mean ± SD with *n* = 4; ***P < 0.001.

The airway epithelium is indispensable to a healthy respiratory tract, acting as the first line of defense against the potential harm of inhaled substances. It is also directly targeted by inhaled toxicants such as those present in CS. We thus tested whether Nur77 expression and activity are altered in HBE cells of COPD patients (COPD HBE cells). Nur77 protein level in COPD HBE cells was significantly lower than that in NHBE cells (Fig 2A), indicating downregulation of airway epithelial Nur77 levels in COPD.

Because activated Nur77 regulates transcription of its target genes by binding to DNA sequences within their promoters, such as NurRE [14, 15, 22], we tested if Nur77-mediated transcriptional activity is downregulated in COPD by electrophoretic mobility shift assay. We found that Nur77’s NurRE binding in COPD HBE cells was reduced compared with that seen in NHBE cells (Fig 2B), consistent with decreased expression (Fig 2A). The Nur77-NurRE complex was supershifted in the presence of anti-Nur77 antibody, verifying the specificity of this interaction (Fig 2B). Similarly, DNA-binding activity of Nur77 was impaired in COPD HBE cells, as measured by Nur77-specific reporter assay (Fig 2C). Together, these data show that both expression and transcriptional activity of Nur77 in HBE cells are diminished in COPD.

### Nur77 knockout exacerbates CS-induced lung inflammation *in vivo*

**Fig 3:**
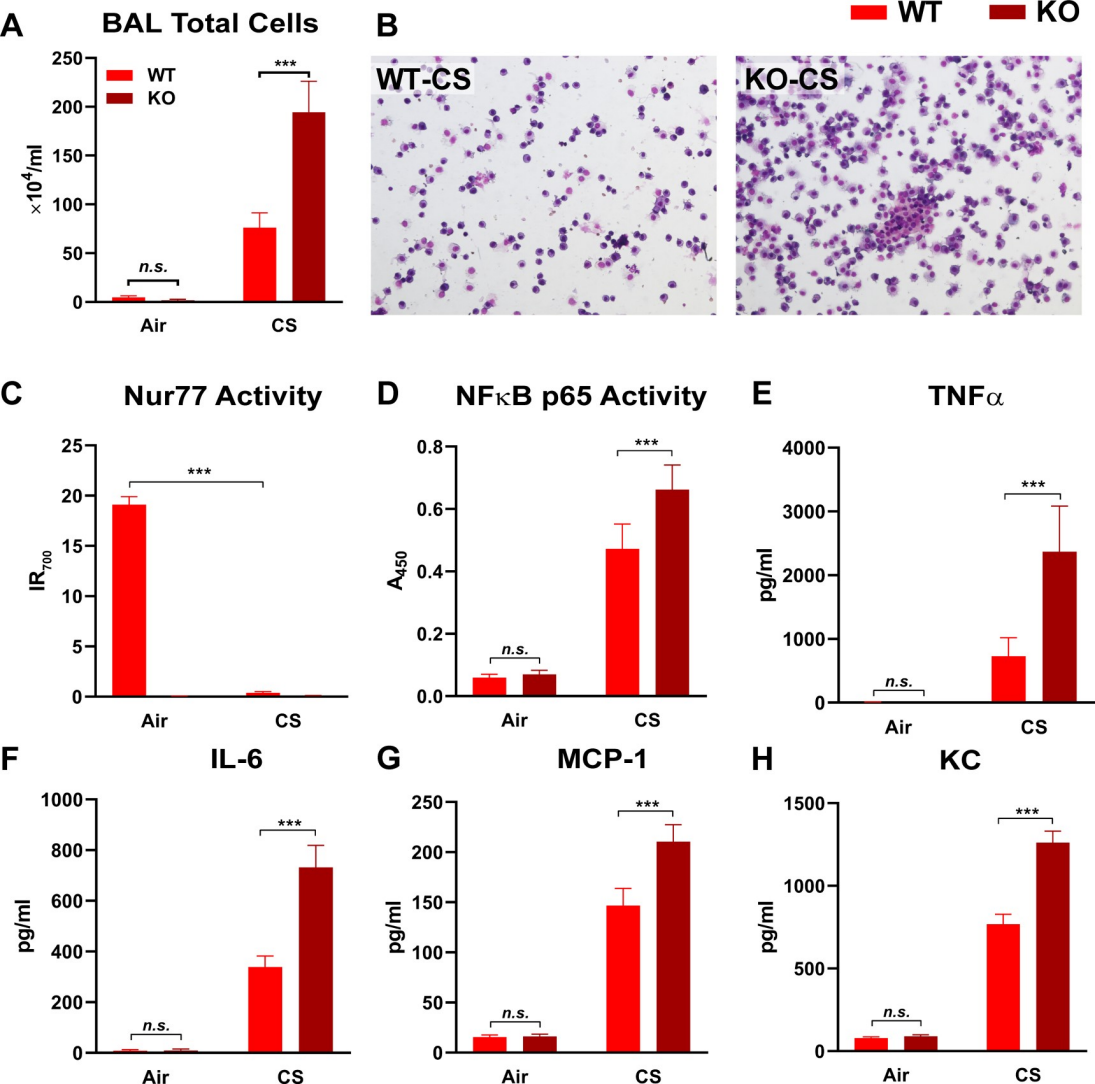
Nur77 knockout increases the levels of CS-induced lung inflammation markers. Inflammatory responses in lung tissue of WT and Nur77 KO mice exposed to filtered air or CS for two months. Total cell counts (**A**) and photomicrographs (20× objective lens) (**B**) of Diff-Quik-stained cells in BAL fluid from the indicated treatment groups. The following markers were measured in lung tissue homogenates by ELISA: activities of transcription factors (Nur77 [**C**] and NF-κB p65 [**D**]) and the levels of cytokines (TNFα [**E**] and IL-6 [**F**]) and chemokines (MCP-1 [**G**] and KC [**H**]). (**C**) Infrared assay endpoint signal (IR_700_) represents Nur77 transcriptional activity. (**D**) Spectrophotometric reading value (A_450_) represents NF-κB p65 activity. (**E**-**H**) The levels of cytokines and chemokines are shown as protein concentrations (pg) in ml of lung tissue homogenates. Data are expressed as the mean ± SD with *n* = 5 mice/group; ***P < 0.001, *n*.*s*. = non-significant.

Cigarette smoking is a major risk factor for COPD, and exposures to the toxicants found in CS elicit lung inflammation [4–8]. To assess if and how Nur77 is involved in such CS-induced inflammation, we compared the levels of Nur77 activity and of several pro-inflammatory mediators in the lungs of WT and Nur77 KO mice in a CS-induced lung inflammation model. Mice were exposed to filtered air or CS for two months. We found that, while CS increased the number of total cells in BAL fluid in both WT and Nur77 KO mice, the increase in the cell count was more prominent in the absence of Nur77 (Fig 3A and 3B). Conversely, CS abolished Nur77’s DNA binding activity in WT mice (Fig 3C). As expected, Nur77 activity was undetectable in filtered air- or CS-exposed Nur77 KO mice (Fig 3C). Conversely, CS exposure caused increased NF-κB p65 transcriptional activity, which was elevated even furthermore by absence of Nur77 (Fig 3D). Likewise, Nur77 deficiency exaggerated CS-induced increases in the levels of pro-inflammatory TNF-α, IL-6, MCP-1, and KC (murine chemokine equivalent of CXCL1) (Fig 3E–3H). Thus, CS downregulated Nur77 transcriptional activity and thereby intensified inflammatory responses in lung tissues of mice.

### CSE downregulates Nur77 via Threonine (Thr)-phosphorylation and aggravates inflammatory responses in human airway epithelial cells

**Fig 4:**
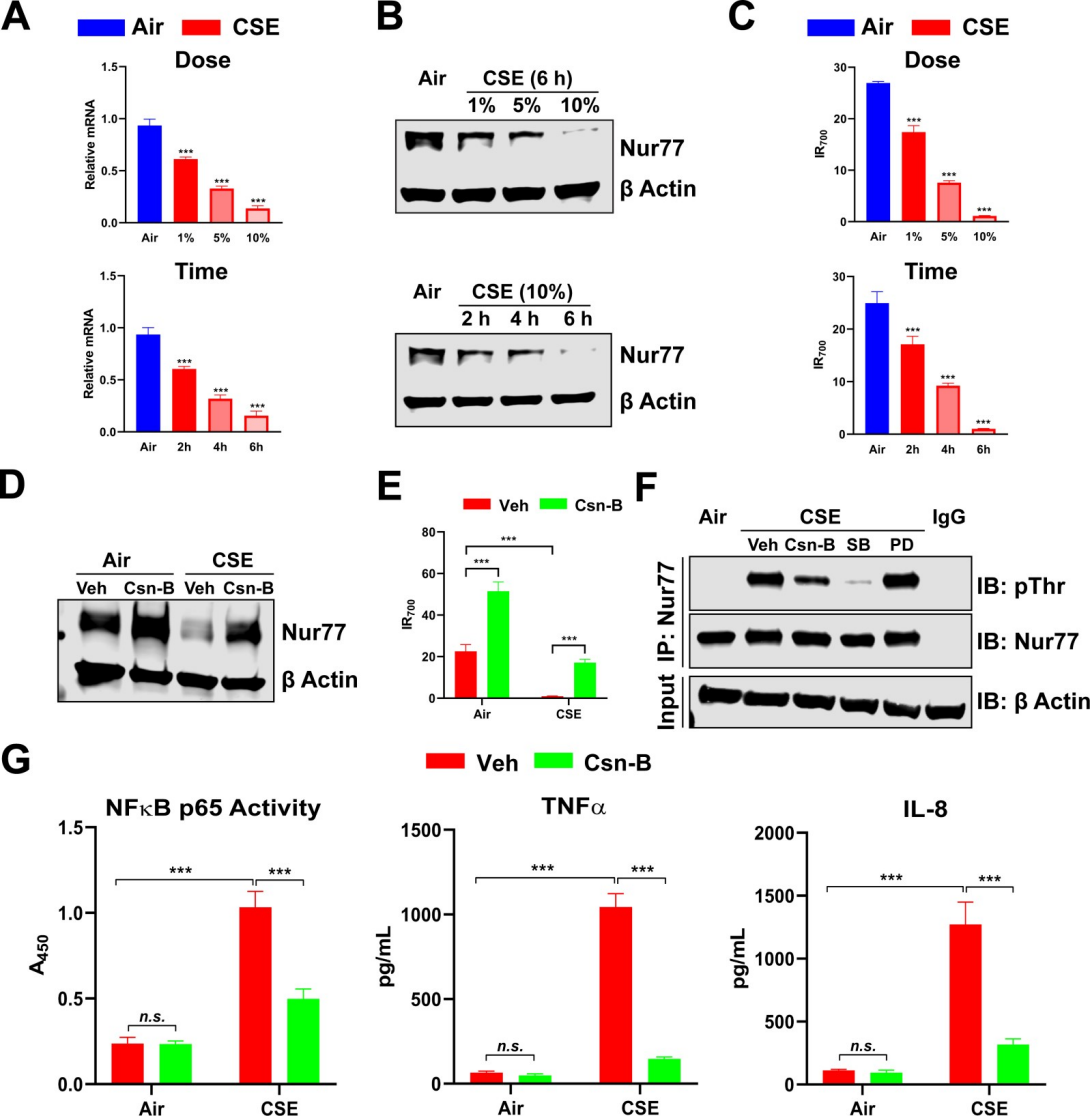
CSE downregulates Nur77 by inducing Thr-phosphorylation and aggravates inflammatory responses in human airway epithelial cells. (**A-C**) Nur77 mRNA (**A**), protein (**B**) expression, and Nur77 transcriptional activity (**C**) in H292 cells exposed to filtered air-treated medium (control medium; Air) or CSE for indicated dose and time, determined by real-time PCR (**A**), Western blotting (β-Actin served as a loading control) (**B**), and NurRE binding assay (**C**). For the dose study, cells were exposed to the control medium or CSE for 6 hours (*upper panel*). For the time-course study, cells were treated with the control medium or 10% CSE (*lower panel*). (**D and E**) Nur77 protein expression (**D**) and transcriptional activity (**E**) in control medium-treated or CSE-exposed H292 cells pretreated with Csn-B (10 μM) or Veh, determined by Western blotting (β-Actin served as loading control) (**D**) and NurRE binding assay (**E**). (**F**) The level of Thr-phosphorylated Nur77 in control medium-treated or CSE-exposed H292 cells, by Western blotting. Cells were pretreated with 10 μM of indicated compounds. SB; the p38 MAPK inhibitor SB203580. PD; the ERK inhibitor PD98059. β-Actin served as an input control. (**G**) Levels of inflammatory response markers (NF-κB p65 activity and TNFα and IL-8 production, by ELISA) in control medium-treated or CSE-exposed H292 cells, pretreated with either Csn-B (10 μM) or Veh. (*left panel*) Spectrophotometric reading value (A_450_) represents NF-κB p65 activity. (*middle and right panels*) The levels of cytokine and chemokine are shown as protein concentrations (pg) in ml of cell culture medium. Data are expressed as the mean ± SD with *n* = 3; ***P < 0.001, *n*.*s*. = non-significant.

To extend our *in vivo* findings and further define the molecular mechanisms of Nur77 downregulation in COPD, we tested the effects of CSE treatment on Nur77 in H292 human airway epithelial cells. In line with our human tissue and *in vivo* mouse model data, CSE reduced both Nur77 transcript and protein levels and its transcriptional activity (Fig 4A–4C). Nur77 was reduced to undetectable levels when the cells were treated with 10% CSE for 6 hours (Fig 4B). Csn-B prevented such CSE-induced reduction in Nur77 expression and its transcriptional activity, restoring them to baseline levels (Fig 4D and 4E).

Nur77 function is regulated by phosphorylation. Because Thr-phosphorylation of Nur77 by p38 mitogen-activated protein kinase (MAPK) was shown to inhibit its ability to suppress NF-κB signaling in a murine macrophage cell line [19], we tested if CSE promotes inhibitory Thr-phosphorylation of Nur77. CSE exposure induced Thr-phosphorylation of Nur77, which was completely absent in control cells exposed to air-treated medium (Fig 4F). Such post-translational modification was partially and completely blocked by pretreatment with Csn-B and the p38 MAPK inhibitor SB203580, respectively (Fig 4F). In contrast, pretreatment with the ERK inhibitor PD98059 had no effect (Fig 4F). CSE exposure elevated the levels of proinflammatory markers including NF-κB p65 activity and TNF-α and IL-8 production in H292 cells, as would be predicted for effects of Nur77 activity-suppressing Thr-phosphorylation (Fig 4G). Csn-B pretreatment inhibited this effect of CSE exposure (Fig 4G). These data suggest that CSE downregulates Nur77 by triggering its Thr-phosphorylation, leading to exaggerated inflammatory responses in H292 cells, whereas Csn-B enhances Nur77’s anti-inflammatory effects by blocking Thr-phosphorylation and preventing loss or degradation of Nur77 activity.

## Discussion

The precise mechanisms by which CS causes COPD are unknown, but voluminous evidence points to inflammation as a key mediating process. Because Nur77 has been found to exert anti-inflammatory actions in other disease models [23], here we tested the ideas that cigarette smoking affects Nur77 and that such action may contribute to COPD pathophysiology. We found that Nur77 is downregulated in human COPD lung tissues, CS-exposed mice, and CSE-treated airway epithelial cells, suggesting that such downregulation is a pathophysiological attribute that may contribute to COPD pathophysiology. Supporting this idea, we found, by assessing agonist-induced Nur77 activation in airway cells, that Nur77 restrains CS-induced inflammatory responses such as those observed in COPD, both *in vitro* and *in vivo*.

Our findings that Nur77 is important in CS-induced, COPD-related airway inflammation are unique but consistent with prior findings related to other diseases. Nur77 deficiency exacerbated OVA-induced allergic airway inflammation in mice [26]. Also, Nur77 expression limited LPS-induced inflammation and tissue damage in a rat model of acute respiratory distress syndrome [27]. Nur77 was identified as a potential modulator of pulmonary arterial hypertension, as its expression was downregulated in lungs of patients with pulmonary arterial hypertension and also in cultured pulmonary microvascular endothelial cells [36]. The evidence thus points to a likely pathogenic role of Nur77 downregulation in multiple inflammatory diseases and to a potential therapeutic benefit of targeting Nur77 pharmacologically to elicit its anti-inflammatory actions.

Contrasting with our findings, Qin *et al*. reported that CS and CSE increased, rather than suppressed, Nur77 in lungs of mice or HBE cells and enhanced Nur77 nuclear export with a concurrent increase in autophagy of the cells [37]. Resolving this discrepancy will require further study, but we speculate that it is attributable to time-dependent differences in CS-induced alteration of Nur77 expression. We assessed short-term CS exposure and found that CS suppressed Nur77 to exaggerate inflammatory responses, whereas Qin *et al*. tested prolonged CS exposure which may preferentially elevate mitochondrial or cytoplasmic Nur77 expression that induces apoptosis/autophagy. Prolonged CS exposure caused Nur77 to bind to Bcl2, which regulates the mitochondrial apoptotic pathway. Because neither our study nor Qin’s compared the compartmentalization of Nur77 under short-term *vs*. prolonged CS exposure, a future time-course analysis may be useful to elucidate the molecular mechanism of Nur77’s anti-inflammatory *vs*. pro-apoptotic functional switch.

Our data that CSE induces Thr-phosphorylation of Nur77 in human airway epithelial cells provide a new mechanism via which CS/CSE triggers Nur77 downregulation. This is an important finding because discovery of a new pathological process offers a novel target for therapeutic interventions. Furthermore, we found that the p38 MAPK inhibitor SB203580 selectively suppressed CS/CSE-induced Thr-phosphorylation of Nur77, suggesting that p38 MAPK is likely the kinase responsible for this post-translational modification. CS activates the MAPK pathway that regulates activities of several transcription factors in multiple cell types [38–41]. Nur77 contains PEST (proline, glutamic acid, serine, and Thr) sequences [42], phosphorylation of which leads to rapid degradation of PEST-containing proteins [43]. Thus, p38 MAPK-mediated phosphorylation may induce proteolytic degradation of Nur77 that contributes to the reduced Nur77 expression we observed. Csn-B also reduced CS/CSE-induced phosphorylation of Nur77 by ~50%, possibly by disrupting the interaction between p38 and Nur77. A synthetic Nur77-specific agonist, *n*-pentyl 2-[3,5-dihydroxy-2-(1-nonanoyl)-phenyl]acetate (PDNPA), similarly disrupted LPS-induced p38 MAPK-mediated interaction and phosphorylation of Nur77, which restored Nur77’s ability to inhibit NF-κB signaling and pro-inflammatory cytokine production [19]. Additional study is necessary to further define the role of p38 MAPK in CS/CSE-induced Nur77 downregulation and the mechanism by which Csn-B restores Nur77 expression.

Subcellular localization represents a key mechanism of Nur77 functional control [23, 44]. Specifically, cytoplasmic/mitochondrial localization is associated with the non-genomic, pro-apoptotic function of Nur77 [44–46], whereas nuclear Nur77 is pro-mitogenic [47, 48]. Extensive inflammation often features tissue damage/cell death due to uncontrolled growth/survival and activities of certain proinflammatory cells. Thus, Nur77’s translocation to mitochondria, which activated p38 MAPK was found to induce [49], precipitates apoptosis of airway epithelial cells and contributes to COPD-associated epithelial loss or injury. It will therefore be important in future work to determine if and how CS/CSE affects translocation, and thus functions, of Nur77. Discovering pharmacological means to rectify any aberrant subcellular localization and functions of Nur77, in a time- or cell type-specific manner, may thus prove translationally relevant for potential COPD therapies.

COPD progression is a key driver of its morbidity and mortality. Because no current therapy effectively prevents disease progression, novel therapies are urgently needed. Therefore, it will be important to further extend our findings of short-term CS exposure effects upon airway inflammation, that often triggers COPD pathogenesis. Future studies using disease models with prolonged (6 to 12-month) CS exposure may provide further valuable insights about Nur77’s role in COPD progression.

## Conclusions

In conclusion, by showing that CS reduces Nur77 expression and activity and that a Nur77-specific agonist can reverse such Nur77 downregulation and counter exaggerated inflammatory responses in the lungs of mice and human airway epithelial cells, our study demonstrates potential therapeutic benefits of Nur77 activation for the treatment of COPD.

## Supporting information

S1 Checklist Completed ARRIVE Guidelines checklist. (PDF)

S1 Table Sequences for primers and oligonucleotides used in the study. (PDF)

S1 Dataset (**Figure A**) Western blot images and densitometry data for Fig 1. (**Figure B**) Western blot, EMSA images and densitometry data for Fig 2. (**Figure C**) Cell count data for Fig 3. (**Figure D**) Western blot images for Fig 4.
(PDF)

## Abbreviations

BAL: bronchoalveolar lavage
COPD: chronic obstructive pulmonary disease
CS: cigarette smoke
Csn-B: cytosporone B
CSE: cigarette smoke extract
DHBE: diseased human bronchial epithelial
HBE: human bronchial epithelial
IL-6: interleukin 6
IL-8: interleukin 8
KO: knockout
MAPK: mitogen-activated protein kinase
MCP-1: monocyte chemoattractant protein-1
Mu: mutated
NHBE: normal human bronchial epithelial
NOR-1: neuron-derived orphan receptor 1
NurRE: Nur-response element
Nurr1: Nur-related factor 1
PD: PD98059
PDNPA: *n*-pentyl 2-[3,5-dihydroxy-2-(1-nonanoyl)-phenyl]acetate
SB: SB203580
Thr: threonine
TNFαA: tumor necrosis factor-alpha
Veh: vehicle
WT: wild type
