# Supplementary material for: Cigarette smoke downregulates Nur77 to exacerbate inflammation in chronic obstructive pulmonary disease (COPD)
Source: PLoS One. 2020 Feb 21;15(2):e0229256. doi: 10.1371/journal.pone.0229256 (PMC7034866; doi:10.1371/journal.pone.0229256)
Supplement: S1 Table — (PDF) [file pone.0229256.s002.pdf]

S1 Table. Sequences for primers and oligonucleotides used in the study.

Gene		Primer Sequence	Tm (°C)	Amplicon Size (bp)
NR4A1 *(Nur77)*	F	5'-CCTGCCAATCTCCTCACTTC-3'	55.4	122
	R	5'-CCAGCATCTTCCTTCCCAAA-3'	55.1	
ACTB (β-Actin)	F	5'-GTGTTGCCCCTGAAGAGCAT-3'	62.5	109
	R	5'-GCTGGGACATTGAAAGTCTCA-3'	60.0	
**Element**		**Oligonucleotide sequence**	**Tm (°C)**	**5'-Tag**
NurRE	Positive	TCAGGTTTCCTCCAAAGGTCA	56.0	IR700
	Negative	AGTCCAAAGGAGGTTTCCAGT	56.0	
