# Supplementary material for: Cigarette smoke downregulates Nur77 to exacerbate inflammation in chronic obstructive pulmonary disease (COPD)
Source: PLoS One. 2020 Feb 21;15(2):e0229256. doi: 10.1371/journal.pone.0229256 (PMC7034866; doi:10.1371/journal.pone.0229256)

Figure A

Full blot image presented in Fig 1 of the manuscript

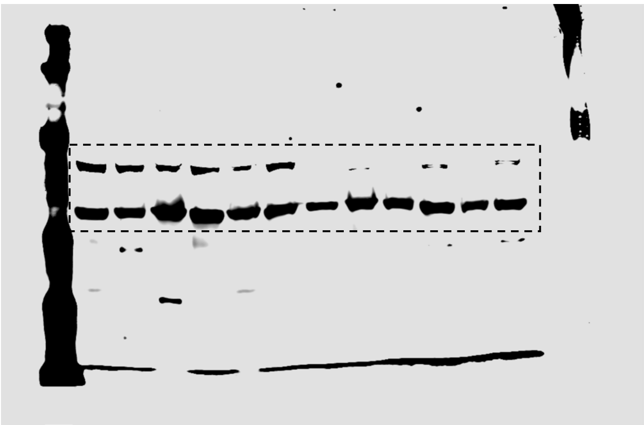

Densitometry

Image Name	Channel	Signal	Area	Bkgnd.	Type	Trim Signal
0000204_01	800	11800	2196	2.13	Signal	9290
0000204_01	800	9140	2196	2.05	Signal	6470
0000204_01	800	4580	2196	1.83	Signal	6890
0000204_01	800	6700	2196	1.86	Signal	5260
0000204_01	800	2410	2196	1.96	Signal	3250
0000204_01	800	8700	2196	2.06	Signal	6290
0000204_01	800	510	2196	1.77	Signal	428
0000204_01	800	1440	2196	1.79	Signal	1120
0000204_01	800	938	2196	1.78	Signal	737
0000204_01	800	2100	2196	1.9	Signal	1600
0000204_01	800	235	2196	2.03	Signal	122
0000204_01	800	2050	2196	1.89	Signal	1610
0000204_01	700	264000	2196	13.3	Signal	252000
0000204_01	700	185000	2196	14.1	Signal	168000
0000204_01	700	331000	2196	26	Signal	302000
0000204_01	700	376000	2196	16	Signal	308000
0000204_01	700	198000	2196	20.1	Signal	111000
0000204_01	700	233000	2196	15.7	Signal	218000
0000204_01	700	143000	2196	9.3	Signal	124000
0000204_01	700	203000	2196	12.8	Signal	193000
0000204_01	700	159000	2196	13.7	Signal	145000
0000204_01	700	246000	2196	12.5	Signal	226000
0000204_01	700	88600	2196	13.8	Signal	80000
0000204_01	700	213000	2196	11.4	Signal	195000

Figure B

Full blot images presented in Fig 2 of the manuscript

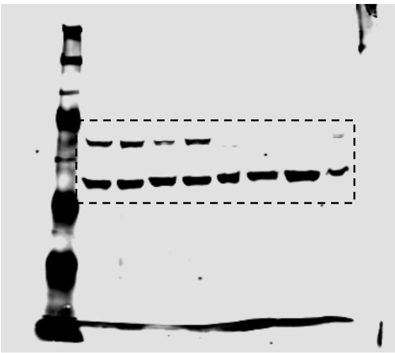

Densitometry

Image Name	Channel	Signal	Area	Bkgnd.	Type	Trim Signal
0000224_01	800	19800	2360	2 68	Signal	14400
0000224_01	800	19500	2360	2.41	Signal	14000
0000224_01	800	12190	2360	2 26	Signal	9880
0000224_01	800	16500	2360	2 35	Signal	11900
0000224_01	800	2320	2360	2 22	Signal	1400
0000224_01	800	1240	2360	2 24	Signal	1050
0000224_01	800	2150	2360	2 22	Signal	1710
0000224_01	800	3540	2360	2.77	Signal	2440
0000224_01	700	73200	2360	7 29	Signal	67100
0000224_01	700	89700	2360	7 66	Signal	80500
0000224_01	700	77300	2360	8 87	Signal	69200
0000224_01	700	99700	2360	7 32	Signal	88500
0000224_01	700	83800	2360	9 04	Signal	70500
0000224_01	700	72700	2360	8 88	Signal	68300
0000224_01	700	79000	2360	10 3	Signal	72000
0000224_01	700	43200	2360	7 05	Signal	43500

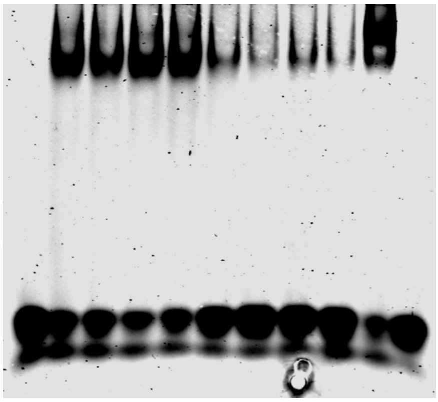

Figure C

Cell count data presented in Fig 3 of the manuscript

CS Model WT and Nur77 KO Mice Cell Counts						
Sample	Sqr I	Sqr II	Sqr III	Sqr IV	Average	Cells/mL
WT-Air Mouse 1	2	3	1	3	2.25	4.5
WT-Air Mouse 2	5	5	2	2	3.5	7
WT-Air Mouse 3	3	3	2	3	2.75	5.5
WT-Air Mouse 4	2	1	3	1	1.75	3.5
WT-Air Mouse 5	2	2	1	1	1.5	3
KO-Air Mouse 1	1	2	3	1	1.75	3.5
KO-Air Mouse 2	2	0	1	0	0.75	1.5
KO-Air Mouse 3	0	1	1	1	0.75	1.5
KO-Air Mouse 4	0	1	1	1	0.75	1.5
KO-Air Mouse 5	0	1	0	1	0.5	1
WT-CS Mouse 1	46	47	33	36	40.5	81
WT-CS Mouse 2	31	28	30	26	28.75	57.5
WT-CS Mouse 3	35	49	37	46	41.75	83.5
WT-CS Mouse 4	31	26	34	36	31.75	63.5
WT-CS Mouse 5	47	53	44	46	47.5	95
KO-CS Mouse 1	84	92	87	112	93.75	187.5
KO-CS Mouse 2	76	85	79	93	83.25	166.5
KO-CS Mouse 3	78	91	89	109	91.75	183.5
KO-CS Mouse 4	122	134	97	127	120	240

Figure D

Full blot images presented in Fig 4 of the manuscript

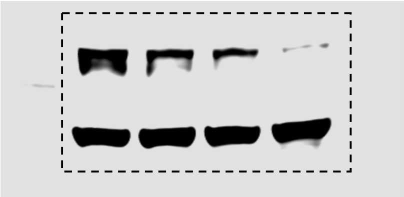

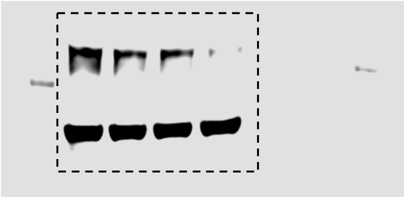

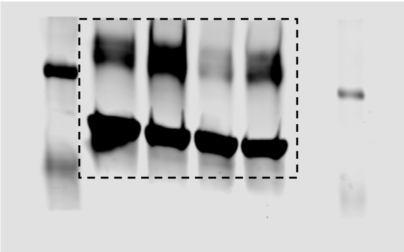

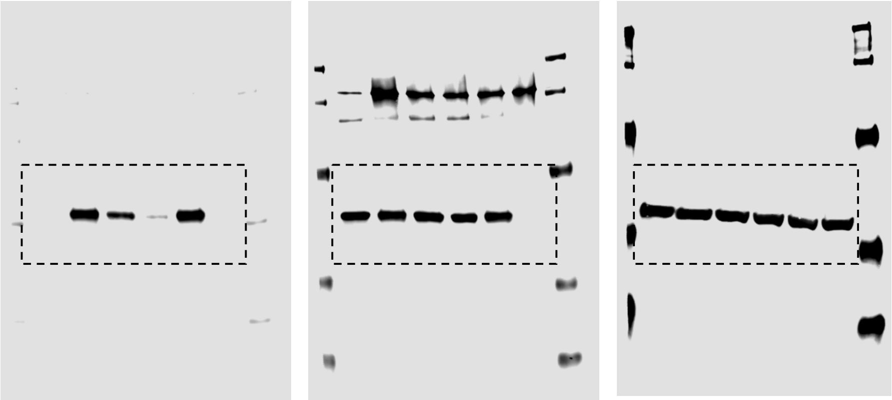

Supplement: S1 Dataset — (Figure A) Western blot images and densitometry data for Fig 1. (Figure B) Western blot, EMSA images and densitometry data for Fig 2. (Figure C) Cell count data for Fig 3. (Figure D) Western blot images for Fig 4. (PDF) [file pone.0229256.s003.pdf]
